# Electrical Transport Mechanisms in Graphene Nanoplatelet Doped Polydimethylsiloxane and Application to Ultrasensitive Temperature Sensors

**DOI:** 10.1021/acsami.2c22162

**Published:** 2023-04-25

**Authors:** Xoan Xosé Fernández Sánchez-Romate, Antonio del Bosque García, María Sánchez, Alejandro Ureña

**Affiliations:** Materials Science and Engineering Area, Escuela Superior de Ciencias Experimentales y Tecnología, Universidad Rey Juan Carlos, Calle Tulipán s/n, 28933 Móstoles, Madrid, Spain

**Keywords:** Graphene nanoplatelets, PDMS, Temperature sensor, Electrical impedance spectroscopy, Electrical properties

## Abstract

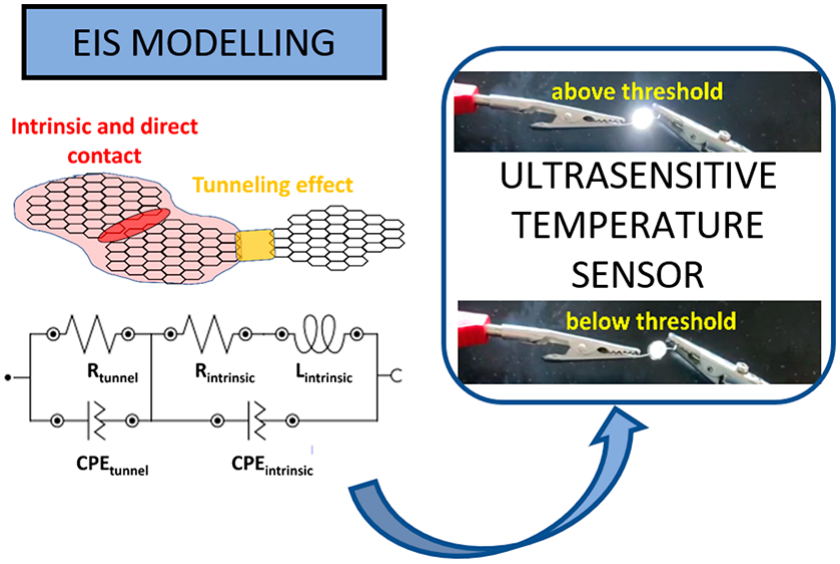

The temperature effect on electronic transport mechanisms in graphene nanoplatelet (GNP) doped polydimethylsiloxane (PDMS) for temperature sensing applications has been investigated under electrical impedance spectroscopy (EIS) analysis. AC measurements showed a very prevalent frequency-dependent behavior in low filled nanocomposites due to the lower charge density. In fact, 4 wt % GNP samples showed a nonideal capacitive behavior due to scattering effects. Therefore, the standard RC-LRC circuit varies with the substitution of capacitive elements by CPEs, where a CPE is a constant phase element which denotes energy dissipation. In this regard, the temperature promotes a prevalence of scattering effects, with an increase of resistance and inductance and a decrease of capacitance values in both RC (intrinsic and contact mechanisms) and LRC (tunneling mechanisms) elements and, even, a change from ideal to nonideal capacitive behavior as in the case of 6 wt % GNP samples. In this way, a deeper understanding of electronic mechanisms depending on GNP content and temperature is achieved in a very intuitive way. Finally, a proof-of-concept carried out as temperature sensors showed a huge sensitivity (from 0.05 to 11.7 °C^–1^) in comparison to most of the consulted studies (below 0.01 °C^–1^), proving, thus, excellent capabilities never seen before for this type of application.

## 1 Introduction

Nowadays, there is a growing interest in the development of new materials with improved multifunctionalities. In this regard, the use of nanotechnology is gaining great attention.

More specifically, nanoparticle-based polymeric composites are now of wide interest. For example, the use of conductive nanoparticles such as silver nanowires or graphitic nanofillers, when added to an insulating medium, such as a polymer matrix, promotes an enhancement of the electrical conductivity of several orders of magnitude at relatively low nanofiller contents.^1−5^ Therefore, it opens a wide range of applications such as, for example, strain sensors,^6−8^ wearable devices,^9−11^ electromagnetic interference shielding,^12−14^ or thermal heaters.^15−17^

The basis for electrical conductivity in nanofilled polymers lies in the fact that the incorporation of these conductive nanoparticles induces the creation of percolating networks that allow the electronic transport via intrinsic and contact mechanisms between adjacent nanoparticles or via a tunneling effect between neighboring ones that are separated by a thin dielectric medium. These percolating networks are sensitive to different stimuli such as chemical agents, mechanical strain, or temperature. For example, the use of relatively low-filled composites generally leads to high sensitivity devices^18^ whereas high-filled ones are promising for applications requiring high values of electrical conductivity^19^ while their sensitivity, especially, to mechanical stimulus is often compromised.

In this sense, numerous efforts have been made to model the electrical properties of these systems by taking several parameters into account such as orientation, waviness, and distribution of the nanoparticles within the polymer matrix, as well as the intrinsic geometry of nanofillers.^20−23^ For example, it has been observed that, for strain sensing purposes, usually graphene is typically much more sensitive than carbon nanotubes due to its 2D nature. The reason for this higher sensitivity lies in the fact that the overlap area between nanofillers is higher in 2D particles and, therefore, the nanofillers can be more separated to form an efficient electrical network as the tunneling transport mechanisms follow a linear-exponential correlation with interparticle distance, promoting an increase in tunneling effects compared to the 1D nature of carbon nanotubes. However, although these models can offer a global view of the electrical behavior of these types of materials, they often fail to explain the accurate contribution of each of the main electrical transport mechanisms in the overall performance. This is explained because the precise contribution of the different conducting mechanisms, intrinsic conductivity of nanofillers and contact between adjacent and tunneling transport between neighboring nanoparticles, is not often given, as it is quite difficult to be measured or quantified.

Moreover, it is very interesting to explore the sensitivity of these materials to temperature, since the electronic transport both in the nanofillers and in the polymer matrix is highly affected by this parameter. In this sense, there are a lot of studies dealing with the determination of the electrical conductivity as a function of temperature.^24−26^ In general, it has been observed that the behavior of the electrical conductivity with temperature strongly depends on the content of the nanofiller. For example, in the case of graphitic nanofillers, some works reported that the electrical conductivity generally increases with temperature at low nanofiller contents, as it is mainly dominated by the electrical transport of the matrix, whereas at higher nanofiller loads, the opposite trend has been observed as there are higher scattering effects in the interface nanofiller–matrix.^27,28^ However, the electrical transport mechanisms significantly depend on the matrix and nanofiller type and the above-mentioned behavior may not be observed.^29^ In fact, several studies have reported a significant decrease in electrical conductivity with temperature in flexible polymers with relatively low nanofiller contents.^30,31^

Therefore, a deeper analysis of transport mechanisms is needed to better understand the temperature effect. For this reason, this study aims to characterize the electrical properties of highly stretchable sensors made with polydimethylsiloxane (PDMS) doped with graphene nanoplatelets (GNPs) under complex impedance analysis. In fact, this technique has been used to characterize the frequency-dependent behavior of the electrical conductivity as a function of nanofiller content^32,33^ or temperature.^34^ These measurements can give very detailed information on main electrical transport mechanisms by incorporating theoretical models which may capture the frequency-dependent behavior of the electrical response.^35,36^ However, the incorporation of these models and their corresponding analysis with temperature remains to be investigated since the equivalent circuits used for fitting have been studied only for room temperature conditions; so, this study aims to shed light into how the different elements may be affected by temperature, as the electronic transport mechanisms change and, therefore, the models may be altered. The aim of using PDMS as the matrix is because of its high flexibility and failure strain, in combination with a relatively low price and fast curing cycles, which makes this resin very useful for industrial applications.

The ultimate purpose of this study is the development of ultrasensitive and highly stretchable temperature sensors by using the knowledge provided by the theoretical approach. Many studies in the literature have reported very high sensitivities for a wide range of temperatures^37^ and applications such as alarm sensors for the detection of fire or high temperatures^38^ or for biological purposes.^39,40^ A simple proof-of-concept test will be carried out to prove the outstanding sensitivity achieved by the developed sensors while a novel perspective of electrical transport mechanisms taking several parameters, such as GNP interactions, distribution, and temperature effect, will be achieved.

## 2 Experimental Section

### 2.1 Materials

GNPs used in this study were *M25*, supplied by *XGScience*. They have an average lateral size of 25 μm and a thickness around 6–8 nm, as given by the commercial data sheet. Their electrical conductivity is ∼10^7^ S/m parallel to the graphene plane and ∼10^2^ S/m in the transversal direction, data given by the producer.

Flexible resin was a silicone elastomer, PDMS, supplied by *Dow* with a commercial name *SYLGARD 184STM*. The viscosity at room temperature is 3500 mPa s.

### 2.2 Manufacturing of GNP/PDMS Nanocomposites

**Figure 1:**
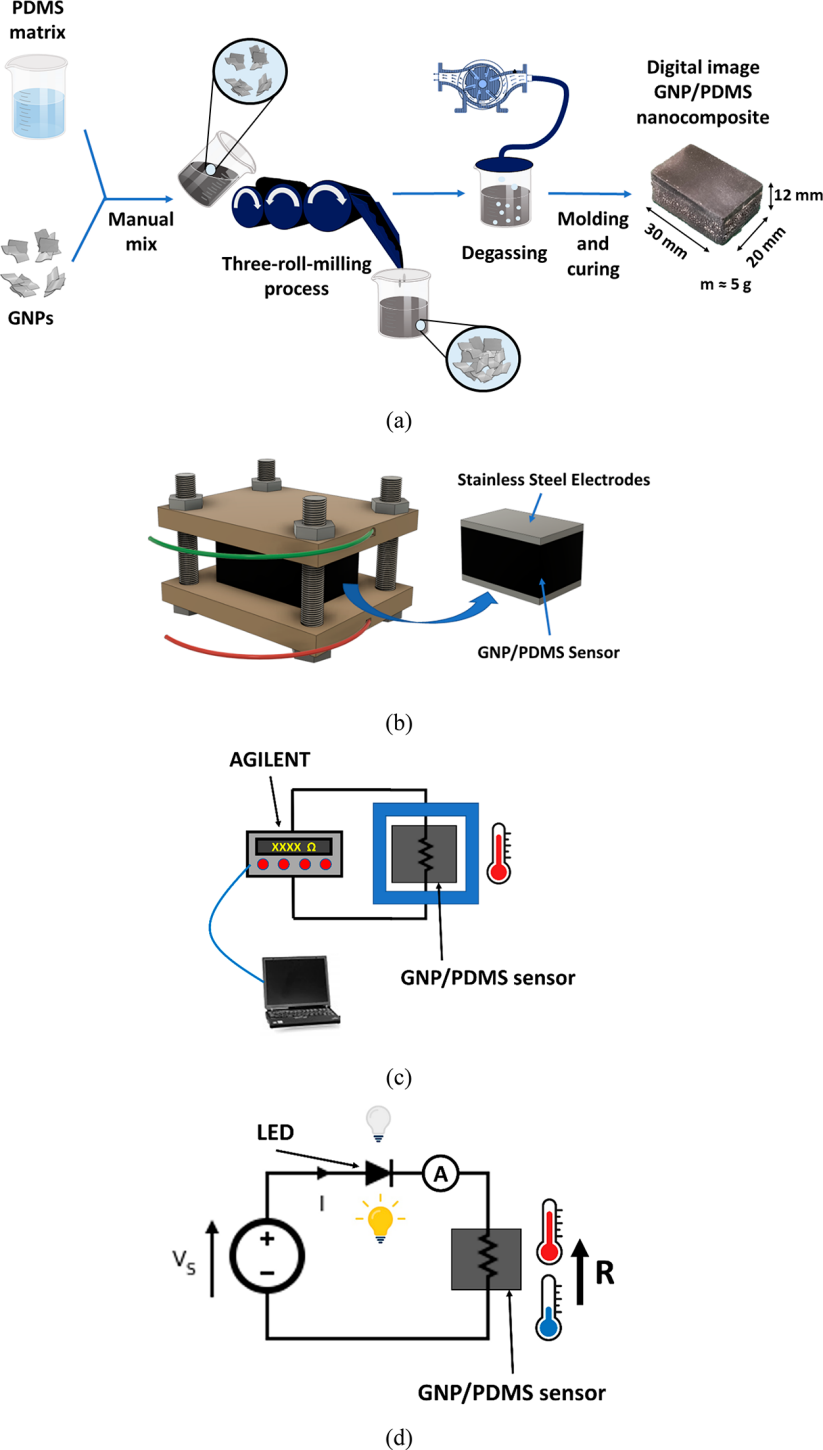
Schematics of (a) the nanocomposite fabrication, (b) EIS measurements under different temperatures, and proof-of-concept tests with (c) DC electrical resistance and (d) LED measurements.

The manufacturing of GNP doped PDMS nanocomposites followed three steps, as shown in Figure 1a: (1) dispersion of GNPs into the PDMS matrix, (2) degassing to remove the entrapped air, and (3) molding and curing.

Dispersion of GNPs was carried out under a three-roll-milling (3RM) process by using a mini-calender *EKATK 80*. The dispersion is achieved by means of shear forces between adjacent rolls rotating in opposite sense and at different speeds: here, a single cycle with a 120 and 40 μm gap between the first–second and second–third rolls, respectively, at a rotating speed of the last roll of 250 rpm, based on a previous study, as a further gap reduction between rolls may promote an early breakage of the GNPs, affecting their electrical properties.^21^

The degassing step was carried out at 100 °C for 10 min using a magnetic stirrer. Subsequently, the curing agent was added in a 10:1 proportion and mixed at room temperature. Finally, the mixture was poured into a Teflon mold and cured in an oven at 120 °C for 25 min, as indicated in the data sheet of the silicone elastomer.

Nanocomposites with 4, 6, 8, and 10 wt % GNP were manufactured. These contents were selected as they are above the electrical percolation threshold of the system, which was found to be just below 4 wt %.^41^ For this reason, the effect of contents near percolation threshold (4 and 6 wt % GNP) and far above (8 and 10 wt %) in the electrical properties as a function of temperature is studied.

### 2.3 Structural Characterization

GNP/PDMS nanocomposites were characterized by X-ray diffraction (XRD) and Fourier transform infrared (FTIR) spectroscopy. X-ray diffraction was done in a *Panalytical X’Pert PRO* diffractometer with a Cu radiation source operating at 45 kV and 300 mA. The scanning of 2θ was carried out from 10° to 90° in substeps of 0.01. FTIR spectra were captured in a Varian Excalibur 3100 apparatus (California, USA) in the wavenumber range of 4000–500 cm^–1^ at 2 cm^–1^ resolution.

The dispersion of GNPs in the PDMS matrix was analyzed by observing cryogenic fractures with scanning electron microscopy (SEM). For this purpose, a Hitachi S-3400N machine was used. The samples were coated by sputtering a thin layer of gold for proper microstructural observation.

Nonisothermal differential scanning calorimetry (DSC) tests were carried out with a *Mettler-Toledo 882e* device from −145 to 70 °C at 10 °C/min. Glass transition temperature (*T*_g_) was evaluated to analyze the influence of the GNP content.

### 2.4 Complex Impedance Measurements

The complex impedance response was studied by means of electrical impedance spectroscopy (EIS) analysis of 30 × 20 × 12 mm^3^ samples in an *AUTOLAB PGSTAT302 N* potentiostat. The samples were sandwiched between two symmetric stainless-steel electrodes and introduced in a PEEK pressure cell to ensure and control the dimensions during the tests as shown in the schematics of Figure 1b.

The AC impedance of the samples was measured at different temperatures (30, 40, 60, and 80 °C) by placing them into a *Carbolite/PN30* convective oven and at a frequency range between 1 MHz and 100 Hz and an amplitude of 30 mV, whereas the temperature of the samples was measured using a thermocouple. Additionally, the electrical properties of the samples with 6 wt % GNP were also measured at 50 and 70 °C. The data was fitted by using different equivalent circuits and *Nova 2.1* software for a proper understanding of the element dependence of the electrical response with temperature.

### 2.5 Proof-of-Concept Test

To prove the capabilities of the proposed GNP/PDMS nanocomposites as temperature sensors, the DC electrical resistance was measured while the sensors were subjected to a temperature variation in an oven. It was measured using an *Agilent DAQ970A* data acquisition system with a *DAQM902A* module at an acquisition frequency of 10 Hz. The aim was to characterize the sensitivity of the developed sensors in terms of electrical resistance change per °C.

The material with the best sensing capabilities was then tested as a temperature alarm sensor. In this test, it was placed in an oven with a thermistor to control the temperature, and it was connected in series to a LED. Here, when the resistance is low, the current flow through the LED is enough to be switched on, whereas in the opposite situation, the LED is switched off. The current passing through the LED was recorded as a function of temperature by using an amperemeter. The schematics of both DC resistance measurements and LED proof-of-concept are shown in Figure 1c,d.

## 3 Results and Discussion

In this section, first, an analysis of the AC electrical response at a fixed temperature is carried out. Then, this behavior will be modeled using an equivalent circuit to better understand the role of each element and how it is correlated to the structure of the material. Furthermore, the effect of the temperature on the electrical properties will be deeply explored in terms of AC measurements and equivalent circuit modeling. Finally, a proof-of-concept to evaluate the temperature sensitivity of the proposed materials will be performed.

### 3.1 Electrical Properties at a Temperature of 30 °C

#### 3.1.1 Electrical Measurements

Before the electrical characterization, it is important to point out that the analysis of XRD and FTIR spectra revealed that the polymer structure does not significantly change with GNP content (Figure S1a,b). More specifically, a similar XFRD spectra can be observed with the characteristic peak at 26.5° in the X-ray diffraction corresponding to the characteristic peak of the graphite crystal plane [002].^42^ In addition, no prevalent differences among the different FTIR spectra are observed for the different GNP contents, showing peaks at 2954 and 1099 cm^–1^ corresponding to the stretching of CH_3_ and Si–O–Si groups; a peak around 1250 cm^–1^ corresponding to the symmetric deformation of CH_3_ group; two peaks at 887 and 796 cm^–1^ that can be stretching vibrations of Si–C and Si–O.^43^ Furthermore, from the DSC analysis, it was also proved that the glass transition temperature was not severely affected by the addition of the GNPs (Figure S1), showing similar values (around −124 °C) for every condition.

**Figure 2:**
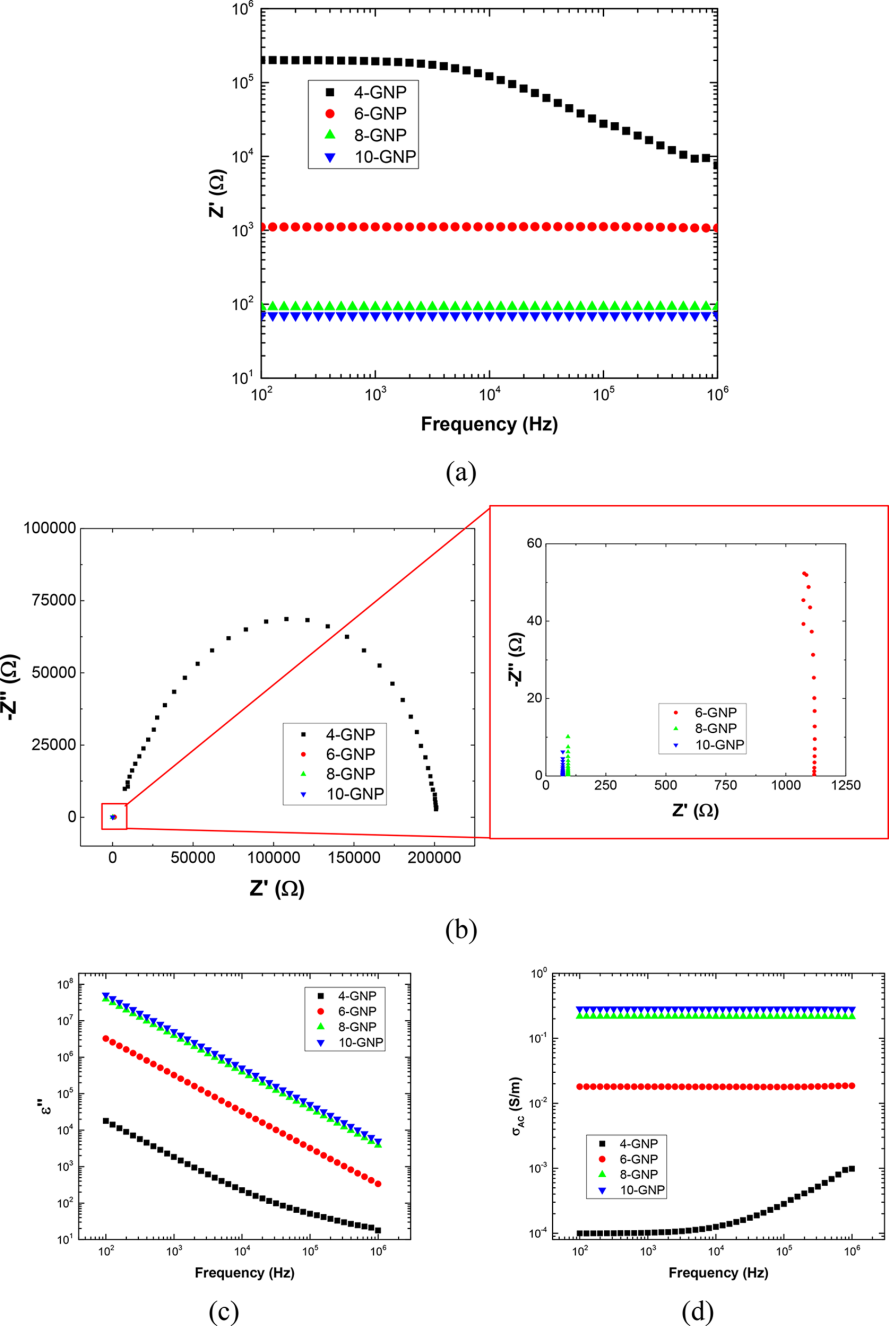
Electrical measurements under EIS showing (a) the real part as a function of the frequency, (b) the Nyquist plots, (c) the dielectric loss, and (d) the AC conductivity for the different conditions.

Concerning the electrical properties, Figure 2a shows the AC measurements for the different conditions, plotting the real part (*Z*′) of the complex impedance as a function of the acquisition frequency. It can be observed that, in the case of 6, 8, and 10 wt % GNP reinforced specimens, the value of the real part is almost constant for the studied frequency range, which means that it is frequency independent. However, in the case of 4 wt % reinforced samples, the value of the real part remains almost constant at low frequencies whereas it decreases at high frequencies. This frequency-dependent behavior is correlated to the density of charge carriers throughout the nanocomposite.^24,44^ At higher GNP contents, the charge carrier density is high enough to travel through the nanocomposite even at high frequencies without any lag between the voltage and the current. However, for lower GNP contents, the charge carrier density is lower and, thus, at lower frequencies the charge carriers can travel through the material, whereas at higher frequencies there is a lag between the voltage and the current. This can be explained by the more prevalent effect of the barrier height of the insulating matrix (PDMS silicone elastomer), as the distance between nanoparticles is expected to be higher as the GNP content decreases.

Here, although the graph of Figure 2a can give some important information, it is necessary to deeply analyze the electrical properties by also studying the frequency behavior of the imaginary part (*Z*″). For this reason, the Nyquist plots are summarized in Figure 2b. Here, it can be observed that, in the case of the 4 wt % reinforced specimens, the Nyquist plot presents a semicircle. It means a high dependency of the electrical properties with the frequency in the measured range, observed for both the real and the imaginary part of the complex impedance response. However, in the case of 6, 8, and 10 wt % reinforced samples, the value of the real part, as previously commented, is almost constant for all the studied frequencies, whereas the imaginary part shows a frequency-dependent behavior. This can be explained because of the formation of several microcapacitors in the material due to both the presence of the nanofillers and the dielectric nature of the polymer matrix.^32,44^

From AC measurements, it is possible to calculate the AC conductivity by following the expression:
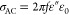
1where *f* is the frequency, ε_0_ is the permittivity of vacuum, and ε″ is the dielectric loss, calculated from the following equations:^45^
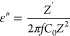
2
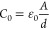
3where *Z′* and *Z* are the real part and the modulus of the impedance, respectively, *A* is the cross-sectional area of the sample (30 × 20 mm), and *d* is the thickness of the specimen (12 mm).

In this regard, the values of dielectric loss and AC conductivity are shown in Figure 2c,d. Here, the previous statements concerning the charge carrier transport are confirmed with a broadening of the DC plateau with an increase in the nanofiller content. As observed before, the AC conductivity did not present any frequency-dependent behavior in the case of 6, 8, and 10 wt % due to the high number of percolating networks created inside the nanocomposite. This is also manifested in an increase of the DC conductivity, obtained from the plateau values of Figure 2d and reflected in Table 1.

**Table 1:** DC Conductivity Values for the Different Conditions

condition	σ_DC_ (S/m)
4-GNP	9.95 × 10^–5^
6-GNP	1.81 × 10^–2^
8-GNP	2.17 × 10^–1^
10-GNP	2.84 × 10^–1^

Therefore, to better understand the role of each constituent in the complex impedance response, an analysis by electrical circuit modeling will be carried out.

#### 3.1.2 Equivalent Electrical Circuit

**Figure 3:**
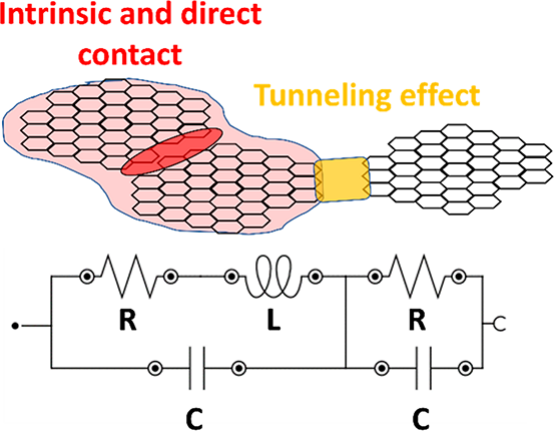
Equivalent circuit showing the LRC (intrinsic and direct contact) and the RC (tunneling effect) elements.

Figure 3 shows a schematic of the general electrical circuit used for simulating the electrical behavior of the nanocomposite. This modeled circuit is based on previous studies,^35,41^ and it is constituted by a parallel RC element connected in series with an LRC element. Here, three main conducting mechanisms can be identified inside the nanocomposites, as explained before: the intrinsic electrical resistance of the nanofillers, the contact resistance between adjacent nanoparticles, and the tunneling resistance between neighboring nanofillers. In this case, the LRC element corresponds to the intrinsic and contact resistance, dominated by the GNP aggregates, that behave as microcapacitors and resistive-inductive elements.^46^ On the other hand, the RC element simulates the electrical properties associated with the tunneling effect, which are dominated by the barrier height properties of the insulating medium.

**Figure 4:**
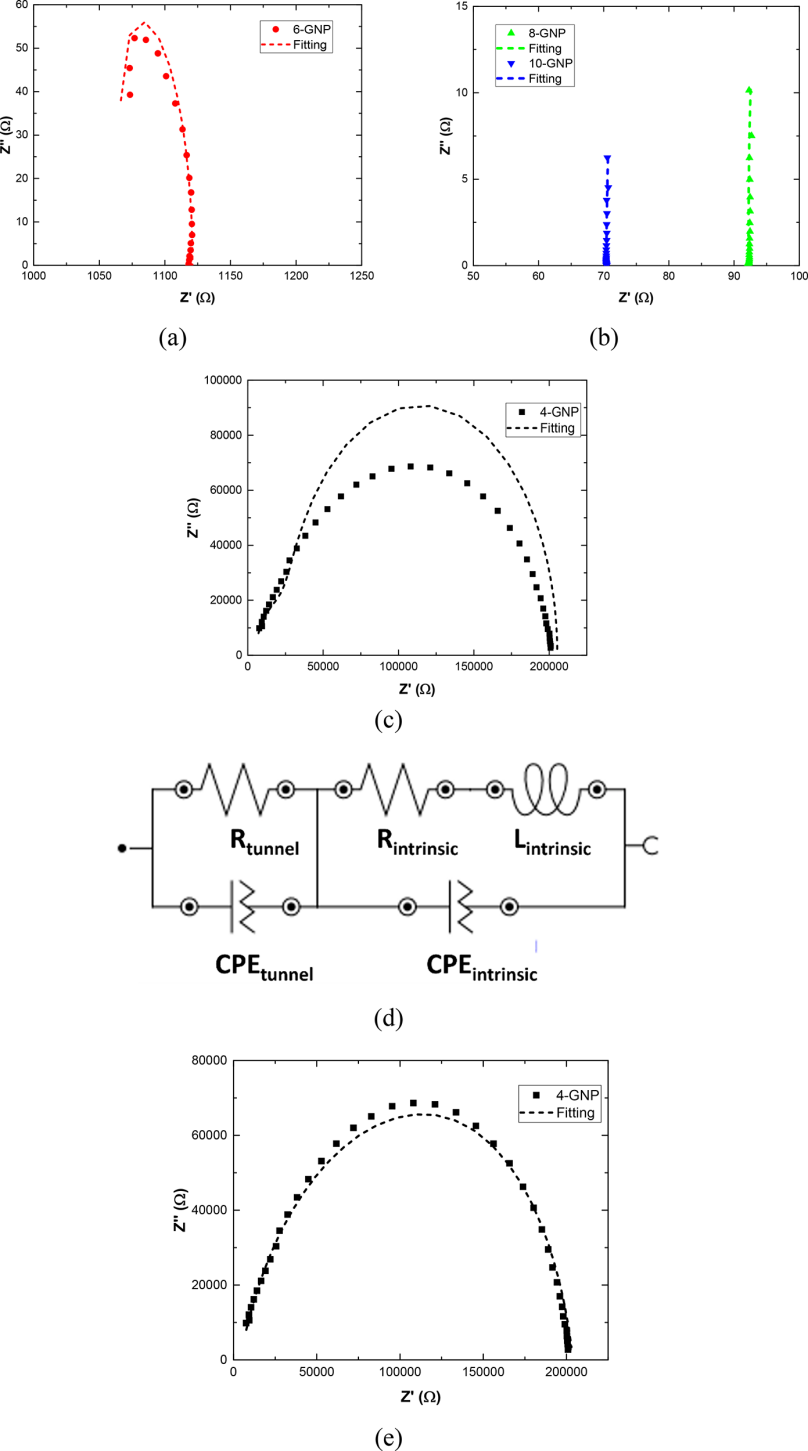
Nyquist plots showing the measured and the fitted data for (a) 6 (b) 8 and 10, and (c) 4 wt % GNP samples using ideal capacitive elements; (d) proposed modified circuit by incorporating CPE and (e) measured versus fitted data for 4 wt % GNP samples using CPE.

In this regard, Figure 4 summarizes the EIS measurements and the fitted results for the complex impedance analysis by using the proposed equivalent circuit. It can be observed that there is a very good agreement between the measured and the modeled results for 6, 8, and 10 wt % reinforced samples (Figure 4a,b). However, in the case of 4 wt % reinforced specimens, significant deviations between the measurements and the theoretical fitting are distinguished (Figure 4c) considering the electrical circuit proposed in Figure 3. More specifically, it can be observed that the complex impedance response does not follow a perfect semicircle but rather a depressed one. This effect has been widely observed in numerous electrochemical studies and is usually correlated to a nonideal capacitive behavior.^47,48^ Moreover, they have been also used in numerous nanostructured composites to properly capture the complex impedance performance.^49,50^ For this reason, the C elements will be substituted, in this case, by constant phase elements (CPEs), as shown in the schematics of Figure 4d.

A CPE represents a capacitive element with a constant phase. Its impedance is defined as *Z* = 1/(*Q*_0_(*j*ω)^*n*^), where *Q*_0_ is a frequency independent factor without physical meaning and *n* is an exponent indicating the capacitive behavior of the element ranging from 0 to 1. A value of *n* = 0 denotes a pure resistive element, while *n* = 1 indicates a pure capacitive element.

The presence of a CPE indicates that there is energy dissipation in the media due to the resistive part of the element, as opposed to a pure capacitor, where there is an effective energy conservation.^51^ In this regard, the results of the fitted circuit including the CPE elements are shown in Figure 4e, where a much higher correlation between the experimental and the modeled results is obtained.

**Figure 5:**
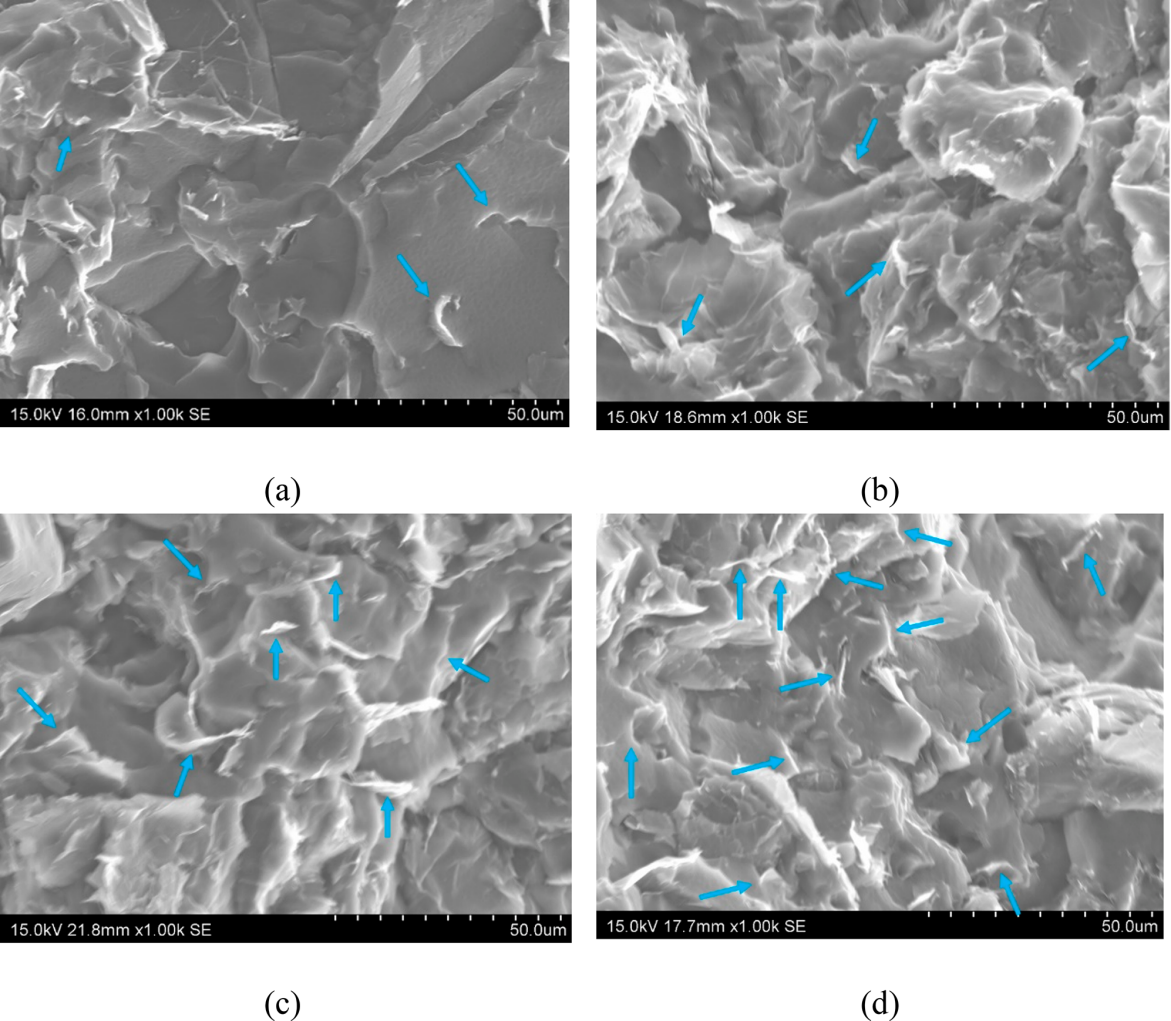
SEM images of fracture surfaces of (a) 4, (b) 6, (c) 8, and (d) 10 wt % GNP samples. GNP domains are marked with blue arrows.

**Figure 6:**
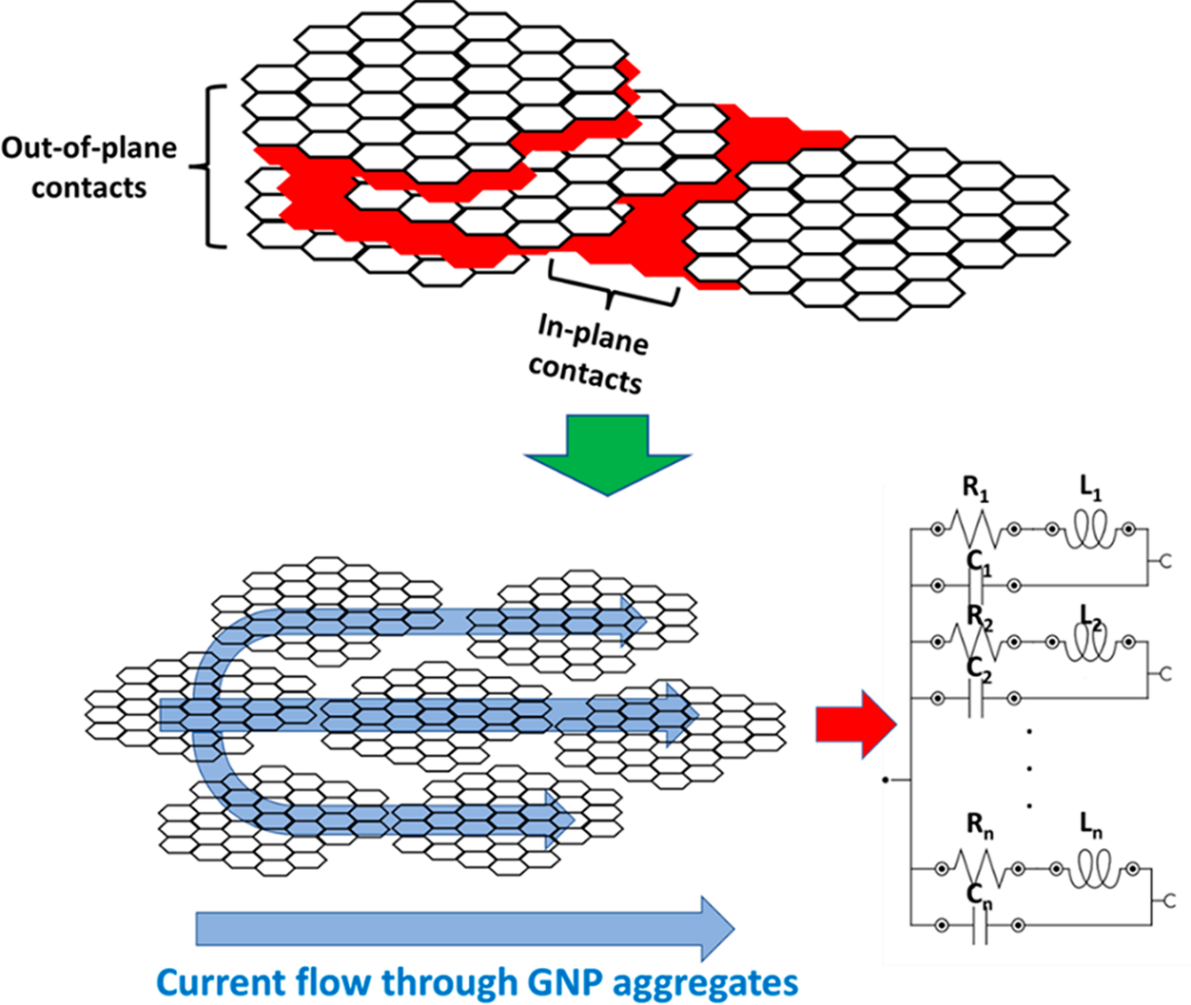
Schematics of electrical transport mechanisms through GNP aggregates highlighting the out-of-plane and in-plane contact mechanisms and their corresponding effect on the equivalent circuit proposed.

Furthermore, Table 2 shows the values of the resistance, inductance, and capacitance elements of the equivalent circuits for the different conditions. From these results, several conclusions can be stated. On the one hand, by focusing on the LRC element, the values of the electrical resistance and the inductance terms decrease as the GNP content increases, whereas the capacitance increases with GNP content. This can be explained by the presence of GNPs distributed inside the dielectric matrix. More specifically, its resistance decreases with the increase of GNP content as the number of contacts between nanofillers increases due to the higher number of nanoparticles. This fact can be observed in the SEM images of Figure 5, where small GNP domains are observed at low GNP contents (Figure 5a,b), whereas these domains are more prevalent when increasing the nanofiller content (Figure 5c,d). In addition, as the GNP content increases, the 2D particles tend to stack in the vertical plane direction.^52^ Therefore, there are more electrical pathways through the aggregates, where the intrinsic and contact resistance are the prevalent mechanisms and, therefore, the equivalent resistance decreases, as shown in the schematics of Figure 6, where the prevalence of out-of-plane mechanisms act in the same direction, as an increase of the number of parallel pathways.

**Table 2:** Values of Fitting Parameters Using the Electrical Circuits Proposed in Figures 3 and 4d

	LRC element			RC element	
condition	*L* (H)	*R* (Ω)	*C* (F)/CPE (n)	*R* (Ω)	*C* (F)/CPE (n)
4-GNP	0.3	9 × 10^4^	–/0.9	1.1 × 10^5^	–/0.75
6-GNP	4.87 × 10^–5^	571	4.86 × 10^–11^	550	1.8 × 10^–10^
8-GNP	2.78 × 10^–7^	2.21	3.58 × 10^–8^	90	2.02 × 10^–10^
10-GNP	1.65 × 10^–7^	1.42	5.89 × 10^–8^	69	2.03 × 10^–10^

Moreover, the same effect is observed in the inductance term, since the presence of several parallel pathways promotes a reduction of the equivalent inductance, according to the following expression:
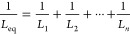
4

However, the opposite behavior is observed in the capacitive term, since a larger number of capacitive elements in parallel promotes an increase of the equivalent capacitance, according to the following expression:
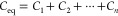
5

More specifically, several studies have shown that increasing the number of GNPs in a dielectric medium (such as PDMS) promotes an increase of the equivalent capacitance of the system.^53,54^

On the other hand, when focusing on the RC element, a similar behavior is observed, with a reduction of the electrical resistance and an increase of the capacitance as the GNP content increases. The reduction of electrical resistance with GNP content is explained according to the well-known Simmons formula for tunneling transport:^55^
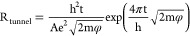
6where *A* is the tunneling cross-sectional area, *e* and *m* are the electron charge and mass, respectively, *h* is the Planck’s constant, φ is the barrier height of the PDMS, and *t* is the tunneling distance between neighboring GNPs.

Therefore, as the GNP content increases, the distance between neighboring nanoparticles decreases, which means that the tunneling distance also decreases. This implies that the electrical resistance associated with the tunneling effect is reduced.^52^

Concerning the capacitance associated with the tunneling effect, it can be modeled following this expression:^35^
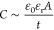
7where ε_0_ and ε_r_ are the permittivity of free space and of the PDMS, respectively.

For this reason, as the tunneling distance decreases, that is, GNP content increases, the capacitance increases, as observed in the obtained values of the fitting circuits of Table 2.

It is important to point out that the PDMS reinforced with 4 wt % GNPs cannot be compared to the other studied materials in terms of capacitance values, as they show nonideal capacitive behavior, as explained before, due to a higher scattering of energy dissipation caused by the presence of less microcapacitors in the system. Here, the most important fact is that the GNP content is too low, so there is a very prevalent energy dissipation and, thus, the electrical properties could be much more affected by the presence of any external stimulus.

As can be seen, the proposed circuit clearly reproduces the electronic behavior of the nanocomposites at a fixed temperature, which makes it possible to distinguish between the electrical transport within aggregates and the electrical transport due to tunneling mechanisms between neighboring nanofillers, as well as the ideal or nonideal capacitive behavior due to scattering effects. Furthermore, the effect of temperature on electrical properties can be deeply explored by using the proposed model.

### 3.2 Temperature Effect on the Electrical Properties

#### 3.2.1 Electrical Measurements

**Figure 7:**
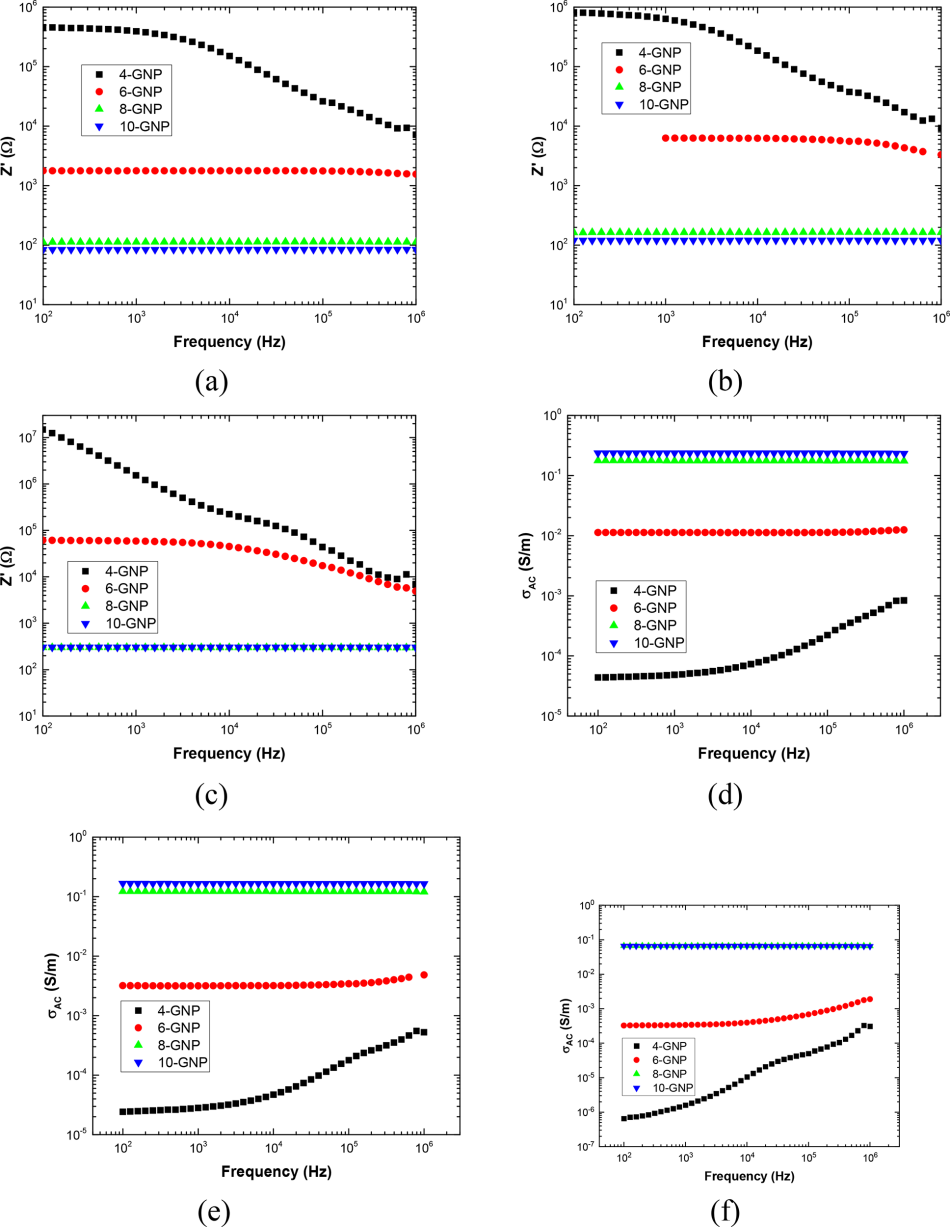
AC measurements showing the real part at (a) 40, (b) 60, and (c) 80 °C and the AC conductivity values as a function of frequency at (d) 40, (e) 60, and (f) 80 °C.

The effect of temperature on the complex impedance response of the studied GNP nanocomposites is shown in the graphs of Figure 7, where the real part of the impedance is represented as a function of frequency. Here, it can be observed that the real part remains almost constant for this frequency range, throughout the temperature range tested for the samples reinforced with 8 and 10% GNP, while those reinforced with 6 wt % present a dependent behavior of the frequency only at 60 and 80 °C (Figure 7b,c). For the material reinforced with 4% by weight, this behavior is clearly dependent on the frequency for all temperature ranges. The same effects are observed when analyzing the AC conductivity values (Figure 7d–f) with a narrowing of the DC plateau when increasing the temperature for the 4 wt % and 6 wt %, which at high temperatures, showed a frequency-dependent behavior of the AC conductivity. In addition, a decrease of the DC conductivity calculated from the AC plateau is also observed with temperature, as reflected in Table 3. Here, in the case of 4 wt % samples, as they did not exhibit any AC plateau for this range of frequencies at 80 °C, the DC conductivity is calculated by fitting the following expression:^56^
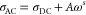
8where ω is the angular frequency, σ_DC_ is the conductivity when ω → 0, *A* is a constant, and *s* is the power law exponent with a typical value in the range of 0.0–1.0.^45^

**Table 3:** DC Conductivity Values as a Function of Temperature for the Different Conditions

	σ_DC_ (S/m)		
condition	*T* = 40 °C	*T* = 60 °C	*T* = 80 °C
4-GNP	4.36 × 10^–5^	2.42 × 10^–5^	4.07 × 10^–9^
6-GNP	1.13 × 10^–2^	3.2 × 10^–3^	3.26 × 10^–4^
8-GNP	1.77 × 10^–1^	1.22 × 10^–1^	6.65 × 10^–2^
10-GNP	2.37 × 10^–1^	1.66 × 10^–1^	6.77 × 10^–2^

**Figure 8:**
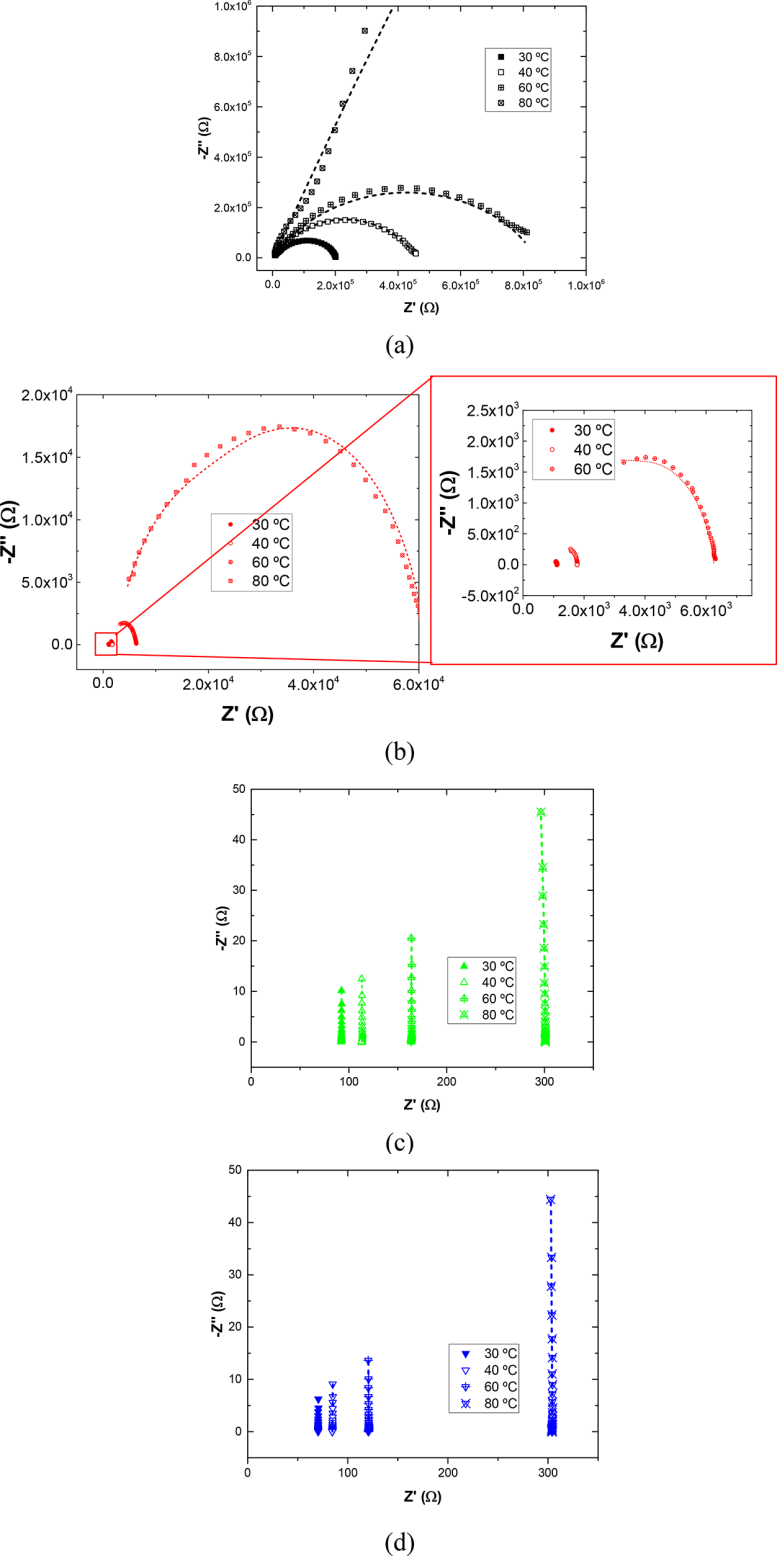
Nyquist plots at 30, 40, 60, and 80 °C for (a) 4, (b) 6, (c) 8, and (d) 10 wt % GNP samples.

The reason for the change in the complex impedance behavior of 6 wt % reinforced samples at high temperatures may be correlated to a decrease in charge carrier density with temperature, which would also affect the DC conductivity values from Table 3 observed, in this case, for every condition. However, to better understand the role of the temperature on the electrical properties of GNP/PDMS nanocomposites, Figure 8 shows the Nyquist plots and the corresponding fittings using the electrical circuits described in Figures 3 and 4d. Here, it can be observed that the proposed circuits also fit the complex impedance behavior very well for this temperature range. Again, for a deeper understanding, the effect of each element of the equivalent circuit will be extensively investigated.

#### 3.2.2 Analysis of Temperature Effect on RC and LRC Parameters

**Figure 9:**
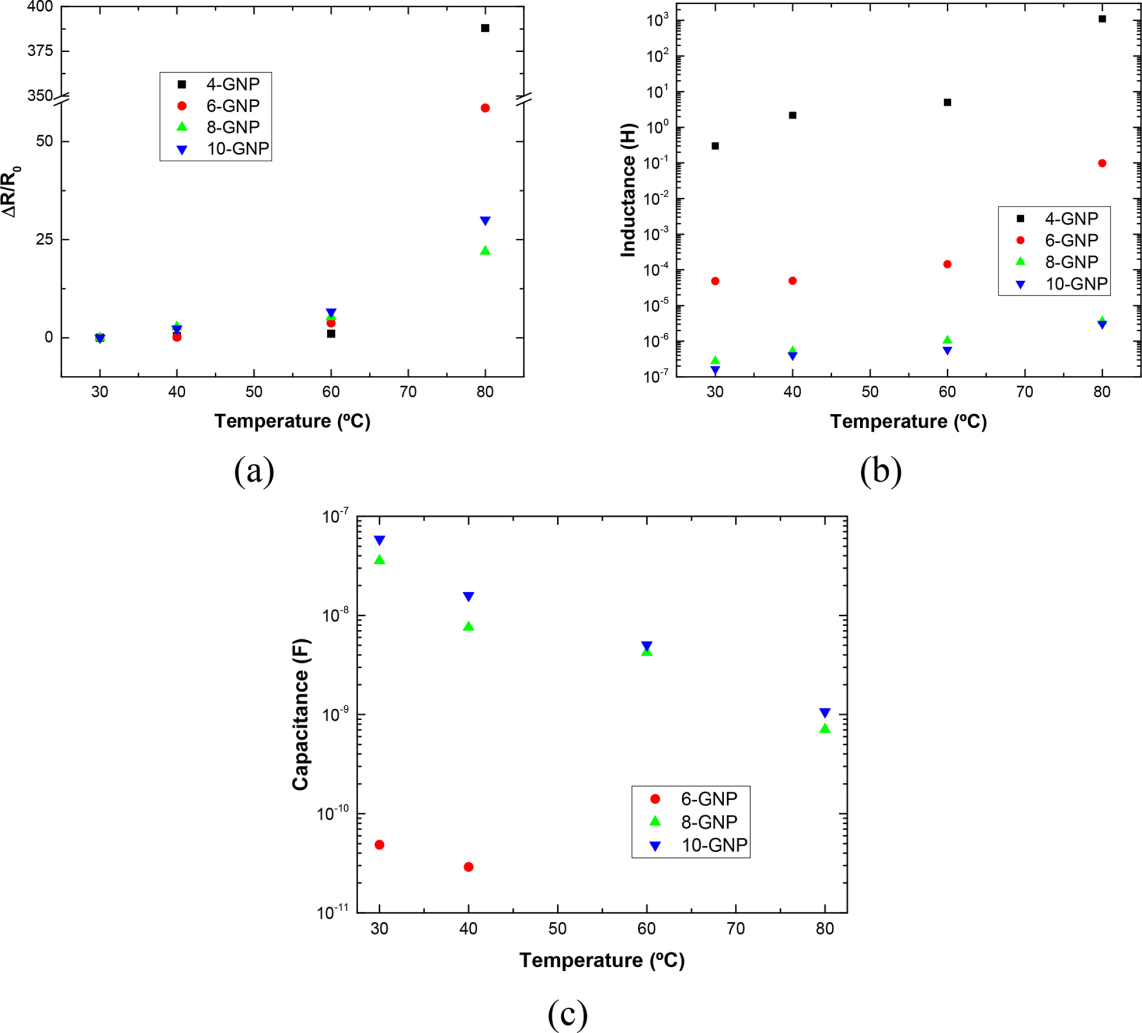
Variations of (a) resistive, (b) inductive, and (c) capacitive element of the LRC part as a function of temperature for the different GNP contents.

**Figure 10:**
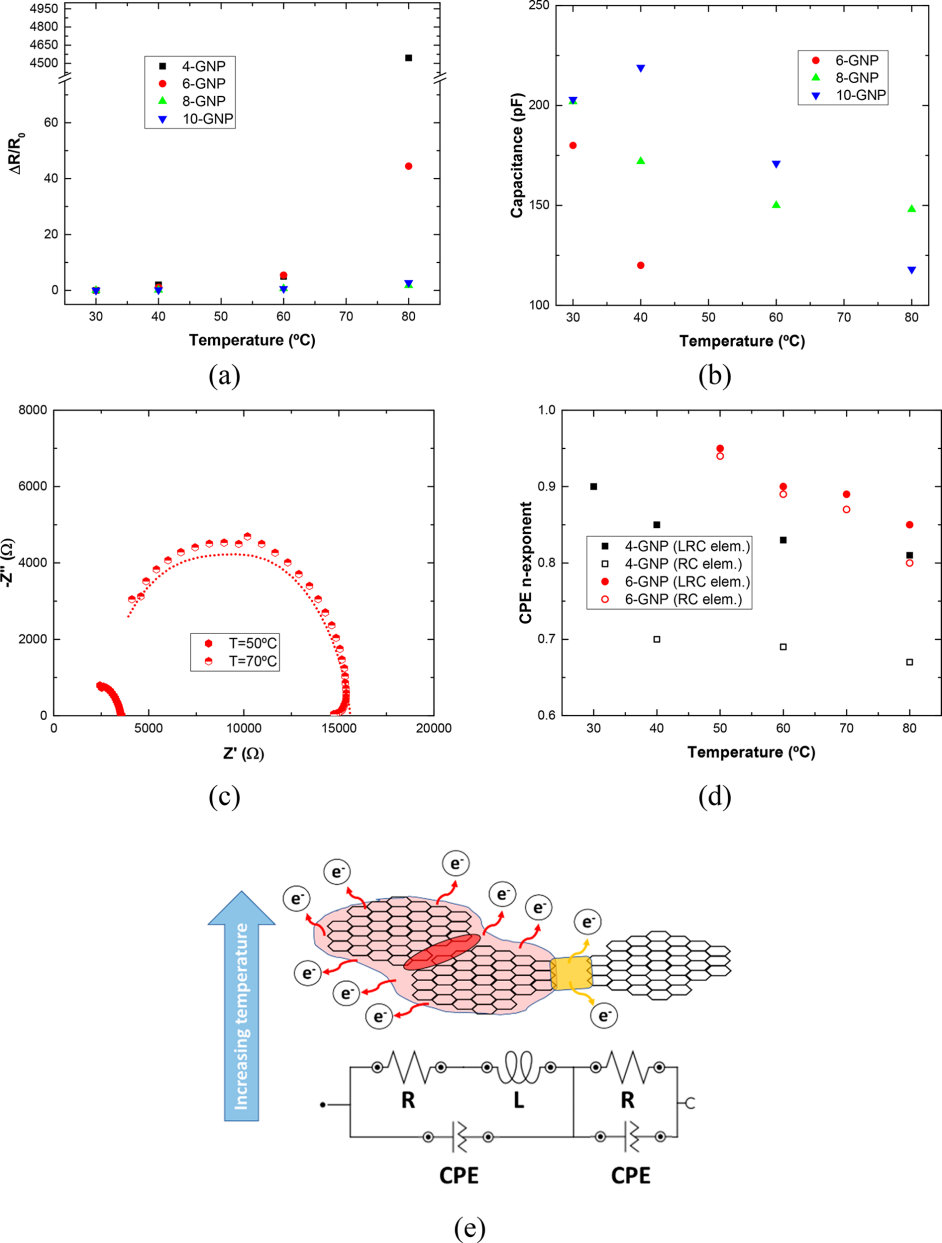
Variations with temperature of (a) resistive and (b) capacitive elements of the RC part, (c) Nyquist plots of 6 wt % GNP samples at 50 and 70 °C, (d) values of *n* exponent of the CPEs as a function of temperature, and (e) schematic of electronic scattering at high temperatures, highlighting the nonideal behavior of the system.

Figures 9 and 10 represent the evolution of the fitting parameters of the circuit proposed in Figure 4d as a function of temperature. Here, it is important to separately analyze the temperature effect on the LRC and RC elements, respectively.

In this sense, on the one hand, the graphs of Figure 9 represent the variation of the intrinsic resistance, capacitance, and inductance of the LRC element. As explained before, it corresponds to the intrinsic and contact mechanisms within nanofiller aggregates. It can be observed that both the electrical resistance and the inductance increase with temperature (Figure 9a,b), whereas the opposite effect can be noticed in the capacitive element (Figure 9c). The reason for the increase in resistance can be found in the fact that, as temperature rises, the kinetic energy of the carriers will be enhanced and, thus, contact resistance will increase due to scattering effects.^57^ Moreover, the metallic nature of the GNPs implies that, by increasing the temperature, the mentioned higher phonon/charge carrier scattering induces a reduction of the mean free path of charge carriers in GNPs causing a decreased mobility and, thus, an increased electrical resistance.^58,59^ These scattering effects, therefore, may reduce the number of contact points affecting the electrical pathways created throughout the agglomerates. This will be reflected in lower parallel pathways and, thus, an increase in electrical resistance compared to room temperature condition but also an increase in inductance and a decrease in capacitance values.

On the other hand, the graphs of Figure 10 summarize the variation of the electrical resistance and capacitance associated with the RC element. As explained before, it is correlated to the tunneling transport mechanisms between neighboring nanoparticles. It can be elucidated that both the electrical resistance and the capacitance follow a similar trend as in the case of the LRC element. However, the explanation for this variation is quite different.

As previously mentioned, the electrical resistance due to the tunneling effect can be modeled according to eq 6. Here, besides the tunneling distance, the height of the potential barrier also plays a very dominant role in the electrical response. Although it is mainly dominated by the insulating medium, it may also be very affected by the presence of the nanofillers, as the interface created between the nanofillers and the insulating medium changes. In fact, many studies have claimed that the presence of local inhomogeneities may promote an increase of the barrier height with temperature.^60,61^ Therefore, according to the expression of eq 6, this will promote a significant increase in electrical resistance following a linear-exponential trend due to tunneling effect, as observed in Figure 10a.

On the other hand, the capacitance is mainly associated with the dielectric properties of the insulating medium. According to eq 7, the capacitance is proportional to the permittivity of the medium, i.e., the PDMS matrix. It has been widely reported that the permittivity of the PDMS decreases with temperature in this range.^59,62^ Therefore, the decrease in the permittivity will promote a reduction of the capacitance associated with the tunneling effect, as observed in Figure 10b.

Furthermore, the fitting parameters of Figures 9 and 10 provide information not only on how the RC and LRC parameters change with temperature and GNP content but also on the ideal or nonideal behavior of the system. More specifically for 6 wt % GNP reinforced samples, the capacitance terms of RC and LRC elements are replaced by CPE at 60 and 80 °C (the capacitance is not represented in the graphs of Figures 9c and 10b). This has been discussed before, and it is correlated with nonideal behavior due to energy scattering effects. Here, for a better understanding of this condition, additional experiments at 50 and 70 °C were carried out for the 6 wt % GNP samples to properly capture the change of the ideal to nonideal behavior. Here, it has been observed that, in both cases, the CPE terms fit the experimental data (Figure 10c), indicating that the nonideal behavior begins at a temperature between 40 and 50 °C. In this regard, Figure 10d shows the value of the exponent *n* as a function of temperature for 4 and 6 wt % reinforced samples. A lower value of *n* exponent denotes, as stated before, a greater resistance effect, that is, a larger energy scattering. Therefore, from Figure 10d, it can be stated that both the decrease in GNP content and the increase in temperature promote higher energy dissipation in the medium due to the resistive part of the element that, in the case of 6 wt % reinforced GNP samples, leads to nonideal capacitive behavior above 50 °C. A schematic of this energy dissipation can be observed in Figure 10e, illustrating the change of the equivalent circuit observed in the case of 6 wt % reinforced GNP samples.

In addition to all the previous facts, it can be observed that the percentage change in resistance due to contact and intrinsic electrical transport mechanisms with temperature is more prevalent in highly filled nanocomposites (8 and 10 wt %) at temperatures below 60 °C, whereas the opposite behavior is found above this temperature. This can be explained because, at higher temperatures, the behavior of 4 and 6 wt % reinforced samples is not ideal, as explained above, with higher energy dissipation and, therefore, higher scattering effects. Regarding the variations of the tunneling resistance, it is found that it is much more prevalent in all the temperature ranges of low-filled nanocomposites due to the combined action of the barrier height of the matrix, which is greatly affected by scattering effects and lack of homogeneity and the tunneling distance between neighboring nanofillers, which is higher at low contents.

Therefore, the proposed equivalent circuit allows a better understanding of the main electrical transport mechanisms in the nanocomposite in a very accurate way. Here, it has been observed that temperature has a very prevalent effect on these electrical transport mechanisms, promoting very significant changes in the electrical behavior of the material, even changing the ideal capacitive behavior of the system. For this reason and due to the extremely high sensitivity of the proposed materials, this study will be completed with the evaluation of their capabilities as temperature sensors.

### 3.3 Proof-of-Concept of Temperature Sensors

**Figure 11:**
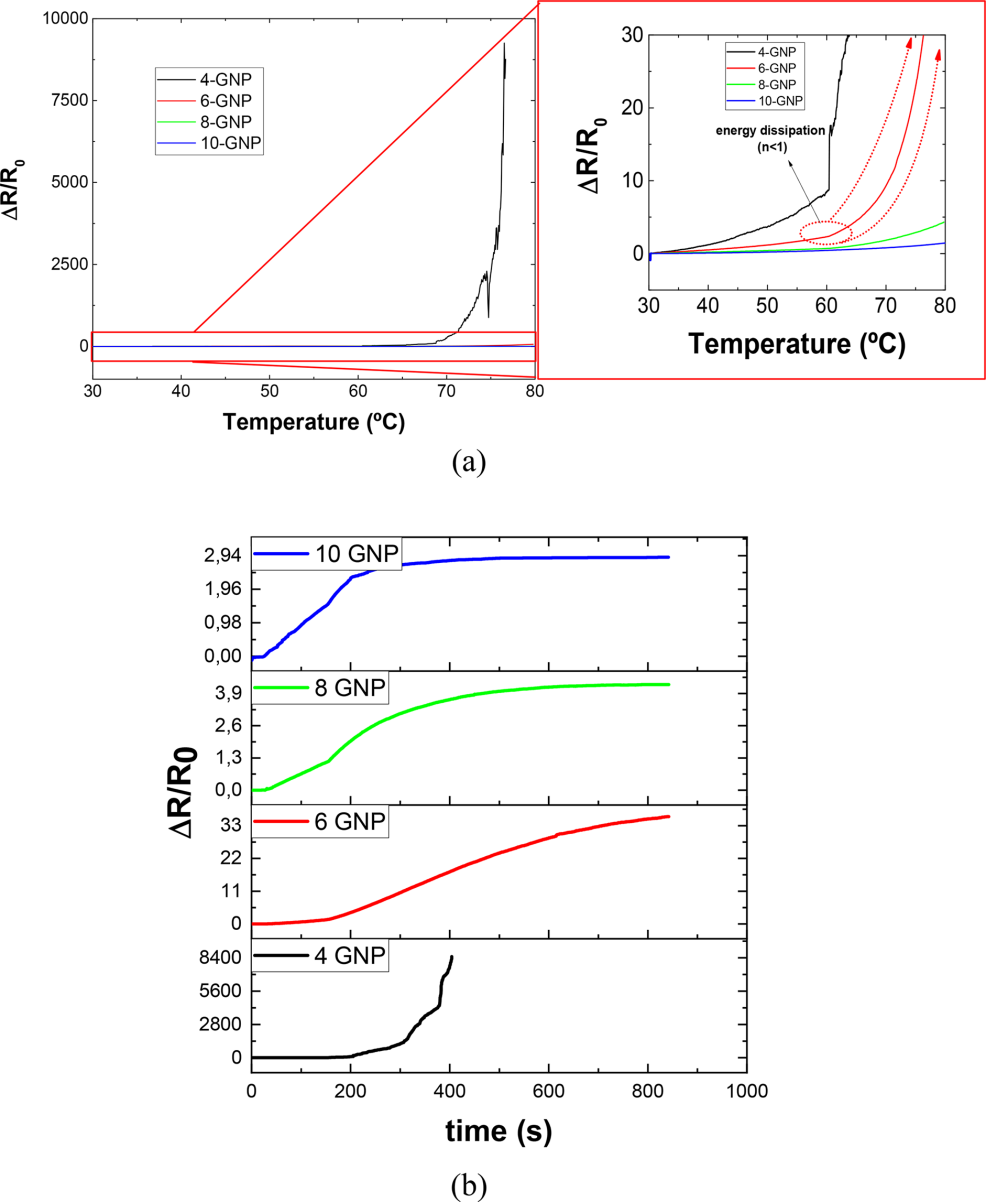
(a) Variation of DC normalized resistance as a function of temperature for the different conditions and (b) DC normalized resistance response at a fixed temperature (65 °C).

As commented before, the ultrasensitive response of the developed materials to temperature makes them very useful for sensing and alarm purposes. In this regard, Figure 11a shows the variation of the electrical resistance under DC measurements as a function of temperature and GNP content. Here, it can be noticed that the electrical resistance increases in a more prevalent way when decreasing the GNP content, as expected from the previous AC analysis.

Furthermore, it can be observed that, in the case of 6 wt % reinforced samples, there is an abrupt change in the behavior of the electrical resistance around 50–60 °C. This can be correlated to the energy dissipation that occurs above these temperatures, which was correlated to a loss of the ideal capacitive behavior (*n* < 1) as shown in Figure 10b. In this case, the energy scattering is reflected in a more exponential increase of the electrical resistance with temperature.

In this regard, it is possible to calculate the sensitivity, α, as the slope (or the derivative) of the resistance–temperature curve given in Figure 11:^63^
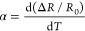
9

Table 4 summarizes the sensitivity values of the different sensors at 35, 50, 70, and 80 °C. Here, as discussed above, it can be observed that the sensitivity increases with temperature. In this regard, the change in the trend for the 6-GNP sensor is observed by an abrupt increase in sensitivity from 50 to 70 °C due to the energy dissipation that occurs through this temperature range.

**Table 4:** Values of the Temperature Sensitivity for the Different GNP Contents at 35, 50, 70, and 80 °C

	α (°C^–1^)			
condition	*T* = 35 °C	*T* = 50 °C	*T* = 70 °C	*T* = 80 °C
4-GNP	0.15	0.204	150	
6-GNP	0.052	0.072	1.47	11.7
8-GNP	0.019	0.023	0.175	0.35
10-GNP	0.0067	0.019	0.054	0.092

However, in all cases, the sensitivity obtained both for a low temperature range (30–50 °C) and for a high temperature one (60–80 °C) is much higher than those observed in other studies developing temperature sensors, as summarized in Table 5. They are also significantly above the standard commercial platinum temperature sensor (0.0039 °C^–1^),^64^ highlighting the applicability of the proposed sensors.

**Table 5:** Comparison of the Sensitivity among Different Sensors Found in the Literature

sensor	α (°C^–1^)	temperature range (°C)	reference
GNP/PDMS (6 wt % GNP)	0.052–0.072	35–50	This work
GNP/PDMS (6 wt % GNP)	1.47–11.7	60–80	This work
GNP/PDMS (8 wt % GNP)	0.019–0.023	35–50	This work
GNP/PDMS (8 wt % GNP)	0.175–0.35	60–80	This work
SWCNT-Ecoflex	0.01	15–45	(63)
Graphene/PDMS	0.008	25–75	(31)
Ag Nanowires	0.0038	25–60	(39)
Graphene/Nitrocellulose	–(0.05/0.03)	140–260	(38)
rGO/Polyurethane	–(0.009/0.0134)	30–80	(40)
Mg film/Ecoflex	0.0024	20–50	(65)
BP/LEG on SBS	0.00174	25–50	(66)
plasma semiconducting SWCNT TFTs	–0.01	36–40	(67)
HiPCO semiconducting SWCNT TFTs	∼−0.012	22–40	(67)

Furthermore, Figure 11b shows the variation of the DC normalized resistance at a response temperature of 65 °C. Here, this variation of the normalized resistance is explained by the response time of the sensor, that is, the time it is necessary to reach a stable DC resistance value when subjected to a fixed temperature. It can be observed that, by increasing the GNP content, the response time is reduced. This is explained by the higher thermal conductivity of the highly filled samples because of the conductive GNP particles.^15^ Therefore, they reach a stable temperature much faster. Here, it can also be observed that the 4-GNP sample lost the electrical contact before reaching the stable temperature due to its low electrical conductivity.

Once the electrical behavior with temperature has been analyzed and the sensitivity of the different materials has been determined, a proof-of-concept as a temperature sensor has been carried out. To achieve this purpose, the sensor was connected in series to a LED. By this way, the changes in the electrical resistance of the sensors will be reflected in changes in the current passing through the LED and, thus, in its luminescence. In this case, only the 6 wt % GNP reinforced sensor was tested. The reason lies in the fact that the electrical resistance of the 4 wt % reinforced one was too high to allow enough current flow through the LED and the 8 and 10 wt % reinforced samples showed lower sensitivities for this purpose.

**Figure 12:**
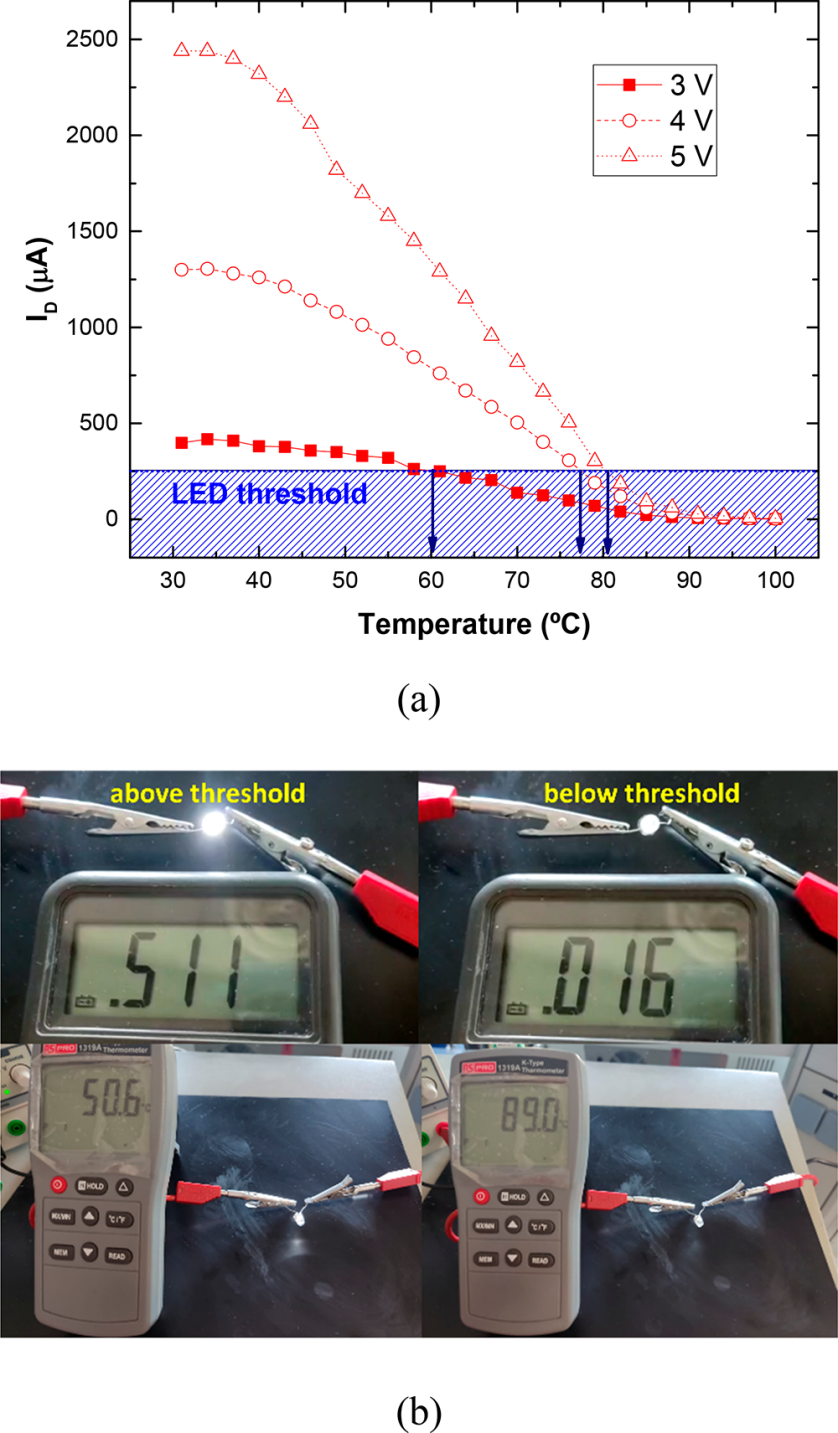
(a) Measurements of the current through the diode, I_D_, with temperature for different output voltages (the diode threshold would act as an alarm at the corresponding temperature) and (b) actual images of the test showing moments above and below the selected threshold.

The results of the temperature sensing test are shown in Figure 12. Here, the current through the LED was recorded by using an amperemeter (as shown in the schematics of Figure 1c). It can be observed that, as the temperature increases, the current passing through the LED decreases (Figure 12a). The threshold for temperature detection can be estimated as a certain value of the current passing through the diode, high enough to achieve proper luminescence. In this case, this threshold has been determined at 250 μA, as it is enough to see the LED properly illuminated. By selecting this threshold, it is possible to obtain the temperature alarm as a function of the output voltage as the intersection of this intensity level with the current–temperature curve of the diode. By this way, the temperature alarms for 3, 4, and 5 V were determined as 60, 77, and 81 °C, respectively (blue arrows in Figure 12a). Therefore, by simply controlling the output voltage, it is possible to establish different threshold temperatures for the same sensor. The proof-of-concept tests were, thus, successful for this purpose with a high sensitivity for temperature determination.

Therefore, the in-depth analysis of the AC and DC behavior of GNP/PDMS sensors aims to understand their behavior against temperature, demonstrating, together with proof-of-concepts, the great potential and applicability of these materials as temperature sensors or alarms.

## 4 Conclusions

The effect of temperature on the electrical transport mechanisms of GNP/PDMS nanocomposites for temperature sensing applications has been explored.

The electrical properties were determined using EIS analysis to have a deeper understanding of how the electronic transport mechanisms occur. In this regard, the electrical equivalent circuit is composed of a RC element in series with a LRC one. It has been observed that the lower the GNP content, the higher is the resistance and inductance and the lower are the capacitance terms. More specifically, with a GNP content of 4% by weight, the material presented a nonideal capacitive behavior and, thus, these elements were substituted by CPE, which means that there is energy dissipation in the system.

The analysis of complex impedance response with temperature showed that the capacitive term decreases whereas an increase of electrical resistance and inductance is observed. This is explained because of the higher scattering effects with temperature, leading to a disruption of electrical pathways that mainly affects the intrinsic and contact mechanisms. Furthermore, the insulating medium is also affected, with a decrease of the permittivity and an increase of the height barrier due to the higher prevalence of local inhomogeneities. In fact, at high temperatures, the samples with 6 wt % in GNP also showed a nonideal behavior, modeled with CPEs, as these local inhomogeneities promote a higher scattering and energy dissipation.

Finally, the proof-of-concept tests as temperature sensors proved an ultrahigh sensitivity in terms of DC resistance variation per unit °C, much higher to those found in the literature. Here, a pronounced linear-exponential effect was found, especially in low filled nanocomposites. Therefore, the applicability of the proposed materials as temperature sensors has been successfully proved.

## Supporting Information Available

XRD analysis, FTIR spectra, and DSC analysis of PDMS/GNP nanocomposites (PDF)
