# Supplementary material for: Electrical Transport Mechanisms in Graphene Nanoplatelet Doped Polydimethylsiloxane and Application to Ultrasensitive Temperature Sensors
Source: ACS Appl Mater Interfaces. 2023 Apr 25;15(18):22377–94. doi: 10.1021/acsami.2c22162 (PMC10176477; doi:10.1021/acsami.2c22162)
Supplement: Supplementary file 1 — am2c22162_si_001.pdf [file am2c22162_si_001.pdf]

Supporting Information for

Analysis of the Electrical Transport Mechanisms in Graphene Nanoplatelet Doped Polydimethylsiloxane by Electrical Impedance Spectroscopy: Application to Ultrasensitive Temperature Sensors

Xoan Xosé Fernández Sánchez-Romate, Antonio del Bosque García, María Sánchez and Alejandro Ureña

Materials Science and Engineering Area, Escuela Superior de Ciencias Experimentales y Tecnología, Universidad Rey Juan Carlos, Calle Tulipán s/n, 28933 Móstoles (Madrid), Spain

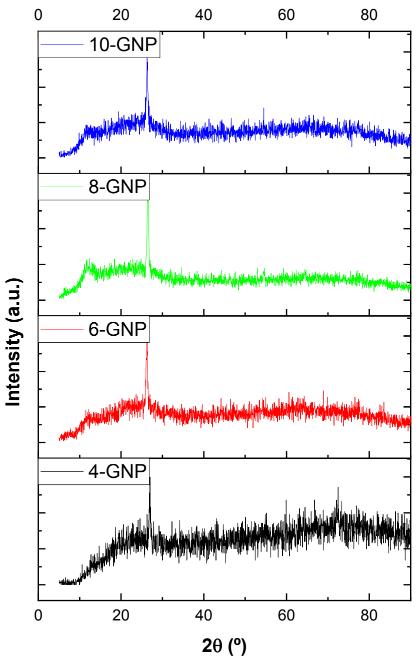

(a)

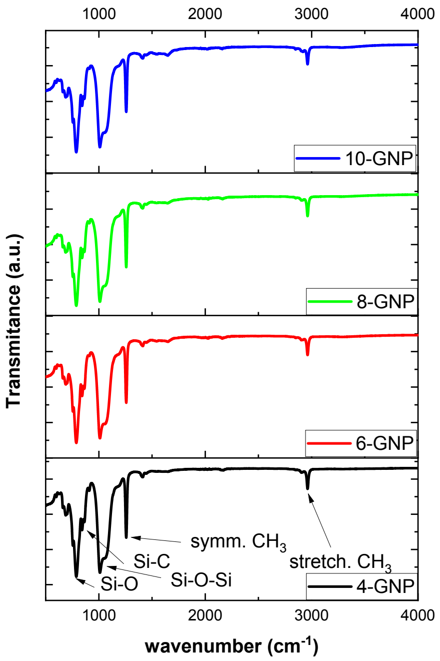

(b)

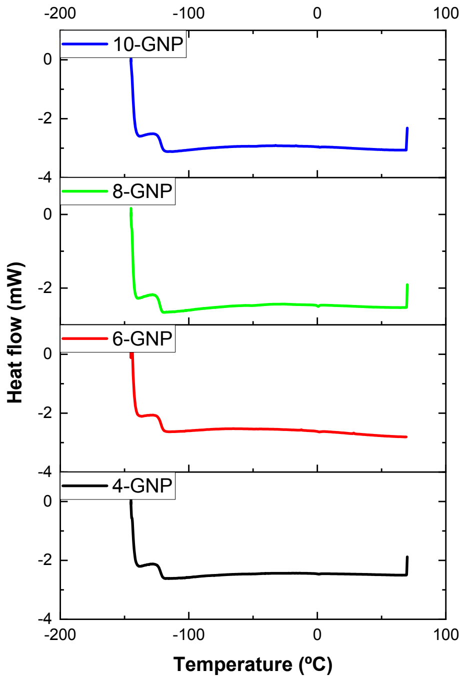

(c)

Figure S1. (a) X-Ray diffractometer (XRD) analysis, (b) Fourier transform infrared spectrometer (FTIR) spectra and (b) Differential Scanning Calorimetry (DSC) analysis of PDMS/GNP nanocomposites.
